# Insight into the emerging role of long non-coding RNAs in BRAF inhibitor resistance in melanoma

**DOI:** 10.3389/fonc.2026.1757030

**Published:** 2026-03-30

**Authors:** Vikas Yadav, Manoj Kumar Jena, Tejveer Singh, Ravi Kumar, Vivek Kumar Garg

**Affiliations:** 1 Department of Translational Medicine, Clinical Research Centre, Skåne University Hospital, Lund University, Malmö, Sweden; 2 Department of Biotechnology, School of Bioengineering & Biosciences, Lovely Professional University, Phagwara, Punjab, India; 3 Translational Oncology Laboratory, Department of Zoology, Hansraj College, University of Delhi, New Delhi, India; 4 Department of Medical Lab Sciences (USAHS), Rayat-Bahra University, Mohali, Punjab, India

**Keywords:** BRAF inhibitor, drug resistance, long noncoding RNA, melanoma, metastasis

## Abstract

Melanoma is the deadliest type of skin cancer, arising from melanocytes, and its global incidence continues to rise. Although multiple somatic mutations contribute towards melanoma development, but the BRAF mutation is the most prevalent. The FDA approval of targeted therapies for BRAF-mutant melanoma greatly increased overall survival in considerable number of patients. However, the majority of patients eventually developed resistance against BRAF inhibitors (BRAFi) and the underlying mechanisms still under explorations. Recent research has revealed that long noncding RNAs (lncRNAs) are closely linked to the development and progression of several malignancies, including melanoma. However, the connection between lncRNA expression profiles and acquired resistance to melanoma BRAFi is in the early stages of research. Herein, we present a current summary of the ways by which lncRNAs either restore melanoma cell susceptibility to BRAFi or cause resistance. Additionally, we have also discussed the potential of lncRNAs role as prognostic indicators of response and resistance to BRAFi therapy in melanoma.

## 1 Introduction

**Figure 1:**
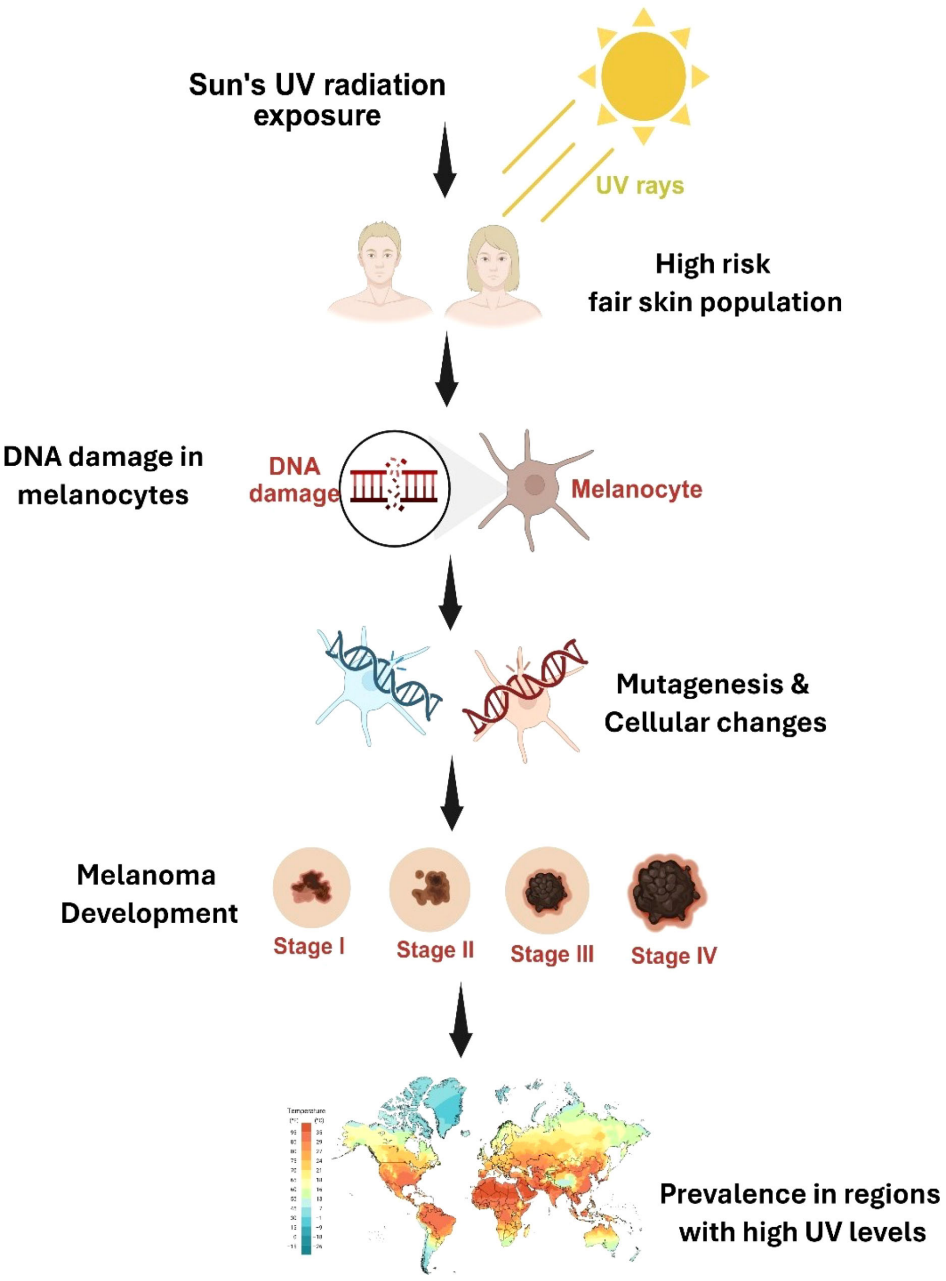
Impact of UV radiation on melanoma development. The figure illustrates the stepwise process by which exposure to ultraviolet (UV) radiation from the sun, particularly in fair-skinned individuals, leads to DNA damage in melanocytes. This damage initiates mutagenesis and cellular transformations, promoting the development of melanoma.

Malignant melanoma is the name for cancer cells that arise from mutations in skin melanocytes (1, 2). Melanocytes are located in the basal layer of the epidermis and produce melanin to protect the skin from photodamage, particularly that caused by ultraviolet (UV) radiation. The etiology of melanoma essentially associated with the aberrant skin cell proliferation enabled by the unrepaired DNA of skin cells which owes to DNA mutations or genetic flaws (2) (**Figure 1**). It is further divided into two categories based on the type of cells involved: basal cell carcinoma (BCC) and squamous cell carcinoma (SCC). Malignant melanoma alone accounted for an astounding 75% of all skin cancer patients’ deaths. Notably, melanoma incidence and death differ by location, with Africa having the lowest rates and USA, UK, and Europe having the highest rates (1, 2). These disparities are influenced by genetic origin, lifestyle, and race. Varied populations have varied melanoma etiology and frequent subtypes. The etiology of cutaneous melanoma, which primarily affects white populations with fair skin, is primarily linked to UV exposure. In contrast, individuals from Asian and African population primarily develop mucosal and acral melanomas, albeit at lower overall incidence rates. Approximately 3,25,000 fresh cases of melanoma were reported in 2020, despite widespread public health campaigns encouraging sun protection (3, 4). Because of aging populations and lifestyle choices that involve intermittent but high levels of UV exposure, the global burden of melanoma is predicted to increase dramatically by 2040. Consequently, melanoma management remains a difficult clinical challenge.

While early-stage melanomas are often treatable with surgery, advanced cases frequently exhibit metastatic spread to organs such as the lungs, liver, brain, and bones, leading to poor prognosis (5, 6). Although effective systemic therapies have significantly improved survival rates, the clinical diversity of melanoma can sometimes delay diagnosis. This delay is particularly problematic as melanoma lesions may resemble benign pigmented spots, making early and accurate diagnosis challenging even for experienced clinicians (5, 6). In addition to alterations in the tumor microenvironment, melanomas are associated with one of the largest burdens of somatic genetic mutations among human tumors (2, 4). These mutations frequently affect genes that regulate central cellular processes, including proliferation (BRAF, NRAS, and NF1), growth and metabolism (PTEN), resistance to apoptosis (TP53), cell cycle control (CDKN2A), and replicative lifespan (TERT), particularly in melanomas arising due to sun-exposed skin under either continuous or intermittent UV exposure (7–9).

**Figure 2:**
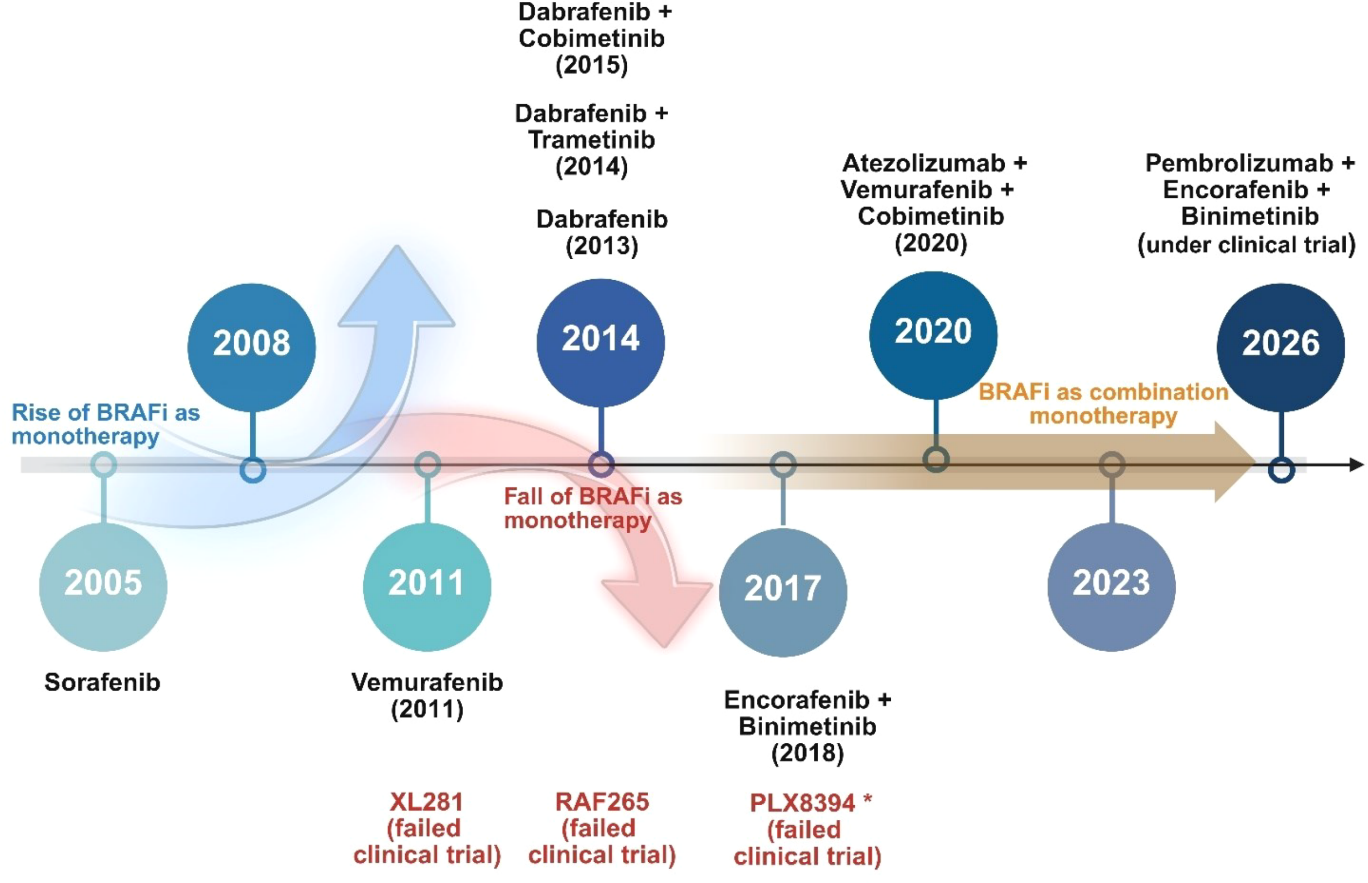
Timeline of rise and fall of BRAFi as monotherapy. The figure outlining the historical progression of BRAFi therapy in melanoma, beginning with the early use of sorafenib (2005), followed by the rise of selective BRAFis such as vemurafenib (2011), marking the emergence of BRAFi monotherapy. The peak of monotherapy was reached around 2011–2013, but resistance development led to its decline by 2014. This was followed by the shift toward combination strategies, notably BRAF and MEK inhibitor pairings. Failed clinical trials (e.g., XL281, RAF265, PLX8394) are marked in red. Ongoing efforts focus on immuno-targeted combinations such as pembrolizumab with BRAF/MEK inhibitors (clinical trial projected for 2026). The figure highlights key transitions from monotherapy to combination and immunotherapy-based regimens to overcome resistance and improve clinical outcomes.

The biology and development of melanoma have been better understood in recent years. It is now evident that there is no single evolutionary pattern that may explain how pre-neoplastic lesions develop into fully evolved melanoma. Different gene mutations, stages of transformation, and precursor lesions are known to be contributors towards evolution into melanoma subtype (1, 10). Proto-oncogene BRAF and NRAS mutations are the primary genetic drivers, and melanomas linked to skin that is exposed to the sun on a regular basis typically have a high mutational burden associated with UV exposure (11). An intriguing discovery is that approximately 80% of benign nevi have a BRAF mutation, which causes limited melanocyte growth by activating cell senescence through oncogene-mediated means. Immunotherapy, BRAF-targeted therapy (**Figure 2**), and modified oncolytic herpes virus are among the therapeutic molecules and combinatorial ways that the FDA has approved since a decade ago (12–14). In with the treatment of metastatic melanoma, the aforementioned therapeutic approaches have shown encouraging results, and several have been approved for use as adjuvant therapies. Compared with standard treatment, BRAF inhibitors (BRAFi) significantly increased response rates and survival in patients with BRAF-mutated melanoma; however, these benefits were short-lived, and the majority of patients developed disease progression due to acquired resistance (11, 15). Consequently, a deeper comprehension of the molecular processes underlying BRAFi-resistance is required to potentially develop more useful treatment approaches and offer more clinical alternatives for melanoma therapy.

Many long non-coding RNAs (lncRNAs) have been functionally linked to human disorders, including cancer, in recent years (16, 17). An emerging theory about lncRNAs states that they are essential transcription regulators during melanogenesis and its metastatic propensity (18). This perspective has resulted in a strong emphasis on clarifying the molecular pathways behind the development of BRAFi resistance in melanoma, even at this stage (19). The existence and synthesis of lncRNAs may be used to gauge transcriptional activity in BRAFi-resistant (BRAFi-R) melanoma. In this review, we have discussed the most recent findings about the mechanisms by which lncRNAs have shown to control immune evasion and BRAFi resistance in melanoma. Additionally, there is discussion regarding the possible predictive utility of circulating lncRNAs for tracking melanoma responsiveness to immunological and targeted therapy.

## 2 The rise and fall of BRAF inhibitors as a monotherapy in melanoma

The multikinase inhibitor sorafenib, which inhibits CRAF, both wild-type and mutant BRAF, as well as several RTKs implicated in tumorigenesis and progression, was evaluated for the treatment of melanoma patients with oncogenic BRAF mutations in an effort to directly target aberrant MAPK pathway signaling (20, 21). However, whether used alone or in conjunction with chemotherapy, sorafenib showed no significant clinical effect in melanoma patients. This lack of efficacy is likely because of its poor affinity for mutant BRAF at therapeutically attainable concentrations (22). Selective BRAFi, such as vemurafenib, dabrafenib, and encorafenib, were created to get around this restriction. These new kinase inhibitors, in contrast to sorafenib, were designed to preferentially bind to the ATP-binding pocket of the active conformation of BRAF, specifically BRAF^V600E^, thereby increasing both efficacy and specificity (23). Vemurafenib received approval from the FDA and EMA in 2011 for the treatment of metastatic melanoma that had BRAF^V600E^ mutations (**Figure 2**). Although some studies indicate that some patients with progressive disease (PD) may benefit from continued vemurafenib treatment after local therapy, the median overall survival (OS) for individuals who took vemurafenib treatment for more than 30 days after local therapy of PD lesions remains unexplored as per our own understanding (24). In contrast, patients who were unable to continue vemurafenib treatment exhibited a median OS of only 1.4 months from the time the disease progression.

## 3 Long noncoding RNAs: biological functions and mechanisms

The long noncoding RNAs (lncRNAs) are typically defined as transcripts longer than 200 nucleotides and can extend up to approximately 100 kilobases in length and are transcribed by RNA polymerase II (RNA Pol II). They possess a poly(A) tail but lack open reading frames (25–27). Mattick et al. and several others have extensively reviewed lncRNAs, revealing that originate from diverse genomic contexts, including promoter regions, enhancer elements, and intergenic loci (28). While some lncRNAs are transcribed from enhancer-associated regions and classified as enhancer RNAs (eRNAs), a substantial proportion of functional lncRNAs arise from promoter-associated transcription units. Importantly, lncRNA function is not determined by promoter or enhancer identity alone, but rather by a combination of factors including transcript sequence, secondary structure, subcellular localization, and interactions with DNA, RNA, and protein partners. In some cases, these interactions involve repeat elements or small interspersed nuclear elements (SINEs); however, a wide range of sequence features, structural motifs, and protein-binding domains contribute to lncRNA-mediated regulated regulatory mechanisms (29, 30). It is known that lncRNAs control transcription in a number of ways: a) they interact with chromatin-modifying enzymes and recruit them at the loci of target genes, b) they interact with heterogeneous nuclear ribonucleoproteins (hnRNPs) to form RNA-protein complexes, which can either promote or repress the gene transcription by recruiting desired proteins to the promoter site or by binding to existing repressors, c) they act as enhancers of transcription by modifying chromatin architecture and recruiting transcriptional proteins to target genes, and thus facilitating transcription process (25, 28, 31). Additionally, certain lncRNAs function post-transcriptionally as molecular decoys for microRNAs or as regulators of splicing, mRNA decay, protein translation, and protein stability.

Some well-characterized lncRNAs have cis-activating function and are transcribed from promoter-associated regions flanked by nucleosomes with modified histones similar to those found at typical promoters, such as histone H3 trimethylated at lysine 4 (H3K4me3). In contrast, enhancer RNAs (eRNAs) are more frequently associated with regulatory regions marked by histone H3 monomethylated at lysine 4 (H3K4me1) (32, 33). However, these chromatin signatures represent general genome-wide trends rather than strict functional determinants, and promoter or enhancer identity alone does not define lncRNA function. Additionally, some cis-regulatory lncRNAs act as allele-specific repressors, for example LncRNA XIST, which regulates the X-chromosome inactivation (33–35). Collectively these studies have led to the conclusion that lncRNAs are extremely diverse and possess a significant degree of functional plasticity, which depends on their capacity to adapt to various molecular interactions and structures as long RNA molecules. Dysregulation of lncRNAs has therefore been implicated in a number of human illnesses, including cancer, cardiovascular and neurological conditions.

## 4 Decisive role of lncRNAs in cancer progression

**Figure 3:**
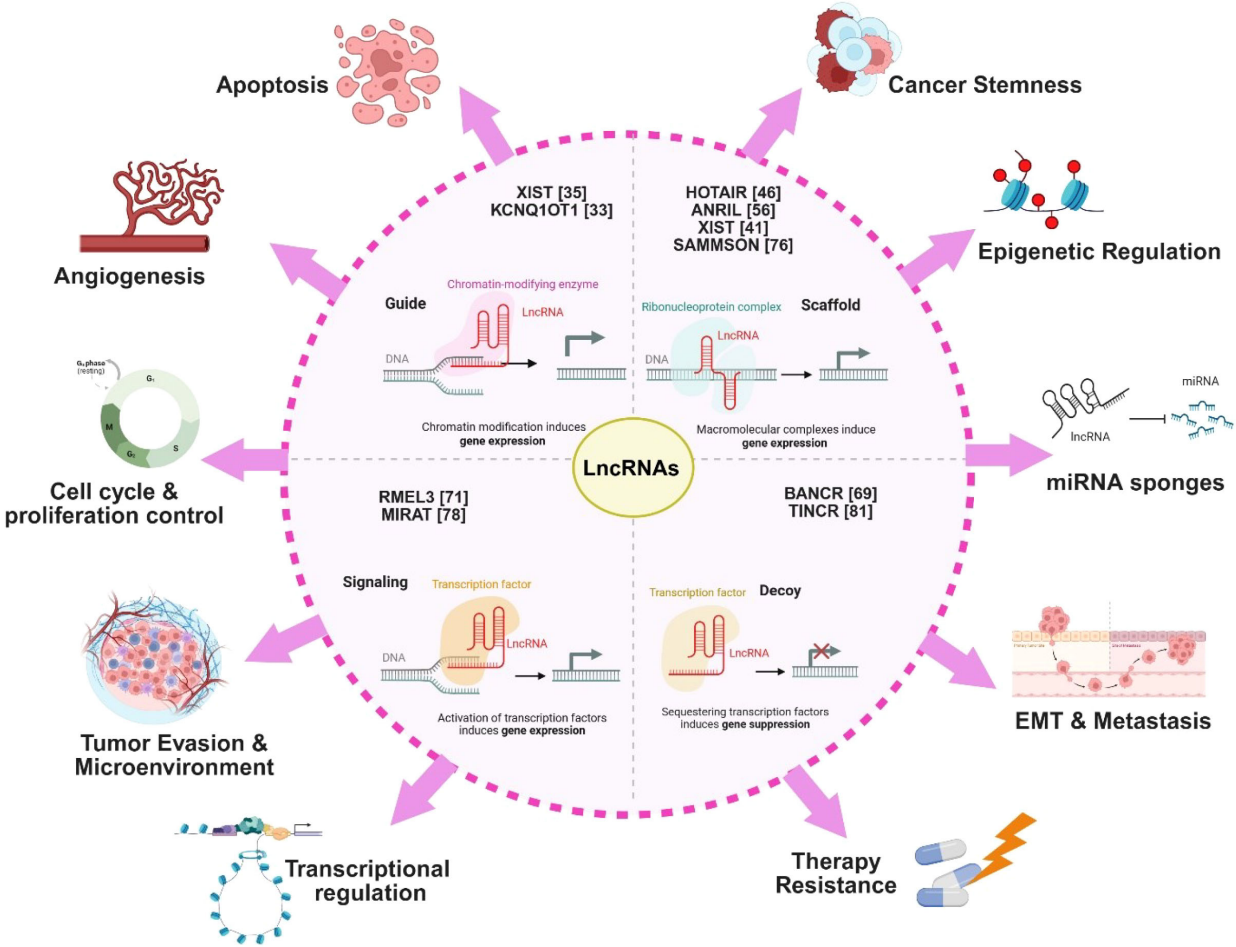
Mechanism & implications of lncRNAs in cancer. Illustration demonstrating the various ways lncRNAs regulate gene expression and physiological processes in human cancers. Each lncRNA and regulatory axis is supported by primary research studies as indicated by reference numbers.

From a molecular standpoint, cancer is a hereditary illness driven by aberrant expression and activity of oncogenes and tumor suppressor genes. The molecular framework of cancers has become increasingly complex with the recognition that, in addition to protein encoding genes, a growing number of lncRNAs functions as oncogenes or tumor suppressors (28, 31). It is widely believed that the functional characterization of lncRNA is currently lagging, despite the quick progress in their discovery and expression analysis. This may be due to the fact that, in contrast to other ncRNAs, lncRNAs are capable of performing wide range of functions. Through direct or indirect interactions with chromatin, proteins, and other RNAs, they can function as decoys, guides, and scaffolds, exerting transcriptional and/or post-transcriptional regulatory activities in the cytoplasm and nucleus (28). As a result, nearly all vital cellular processes are mediated by lncRNAs, both in healthy and diseased states (**Figure 3**). A plethora of literature exists discussing how these lncRNAs contribute to cancer hallmarks and how they are aberrantly expressed in malignancies (**Figure 3**) (25, 36–38). Briefly, the lncRNA FAL1 was observed to have copy number amplification in around 30% of ovarian cancer cases, while lncRNA SAMMSON showed amplification in about 10% of melanoma cases (39, 40). In contrast, loss of lncRNAs XIST1 was observed in significant number of ovarian cancer patients (41). We and several others have recently reviewed more such instances of lncRNAs that have shown their biomarker and therapeutic implications during cancer progression such as LINC00261, MYOSLID, TP53TG1, ACTA2-ASF1, etc (38, 42, 43). Overexpression of several lncRNAs has been observed in the advanced cancer cases associated with metastasis; for instance, MALAT1 overexpression has been reported in metastatic lung adenocarcinoma and colorectal cancer (44, 45). Similarly, breast cancer growth and metastasis have been linked to increased expression of the lncRNA HOTAIR (46). Multiple meta-analyses and systematic reviews have critically highlighted numerous lncRNAs implicated in cancer metastasis (44, 47, 48).

LncRNAs are also known to participate in chromatin remodeling by interacting with various chromatin-modifying enzymes and ATP-dependent chromatin remodeling factors (49). Through a variety of epigenetic mechanisms, including direct interactions with chromatin remodeling complexes, these lncRNAs control the expression of genes. To date, more than 50 lncRNAs have been identified as regulators of these complexes, thereby influencing gene expression, DNA replication and repair processes (50). Chromatin remodelers and lncRNAS are closely associated with cell division, more specifically, play critical role in cytokinesis, thereby impacting cellular differentiation, development, and disease (51). The interaction between lncRNAs and the chromatin remodelers, such as SWI/SNF complex, can either promote or suppress cancer progression depending on the molecular context (52). For example, prostate cancers overexpress the lncRNA *second chromosomal locus associated with prostate 1* (SchLAP1), which has been demonstrated to bind to hSNF5 and counteract the tumor-suppressive action of SWI/SNF complex, hence encouraging tumor invasion and dissemination (53, 54). The detailed epigenetic-based regulatory activity of lncRNAs concerning development and progression of cancer has recently been studied by several authors (55–57).

Dysregulated expression of microRNAs (miRNAs) and lncRNAs has been identified as a hallmark of cancer progression and as an independent prognostic indicator of cancer patients (58, 59). Numerous investigations have demonstrated a close connection between miRNA and lncRNA in controlling RNA transcription. By sequestering miRNAs, lncRNAs can function as miRNA decoys, which will compete with endogenous RNAs and cause the re-expression of miRNA target genes (60). In addition, lncRNAs can compete with miRNAs for specific binding sites within the non-coding regions of mRNAs, leading to enhanced gene expression by preventing the miRNA-induced translational repression (58, 59). Some lncRNAs can be converted into miRNAs, as evidenced by the strong interactions between various non-coding RNA types. The miRNA-lncRNA interaction regulates many cellular functions like proliferation, differentiation, and cell death, proving to be vital in cell physiology (61). Moreover, lncRNAs can be direct target of miRNAs, and small RNAs are generated from lncRNAs during this interaction (62). The miRNAs which target lncRNAs, produce small interfering RNAs (siRNAs) or phased small interfering RNAs (phasiRNA) which then target the downstream genes for their regulation (63). The intricate relation between miRNA and lncRNA has been thoroughly reviewed by several authors (60, 64, 65).

## 5 Emerging role of lncRNAs in the acquisition of BRAF inhibitor resistance in melanoma

**Figure 4:**
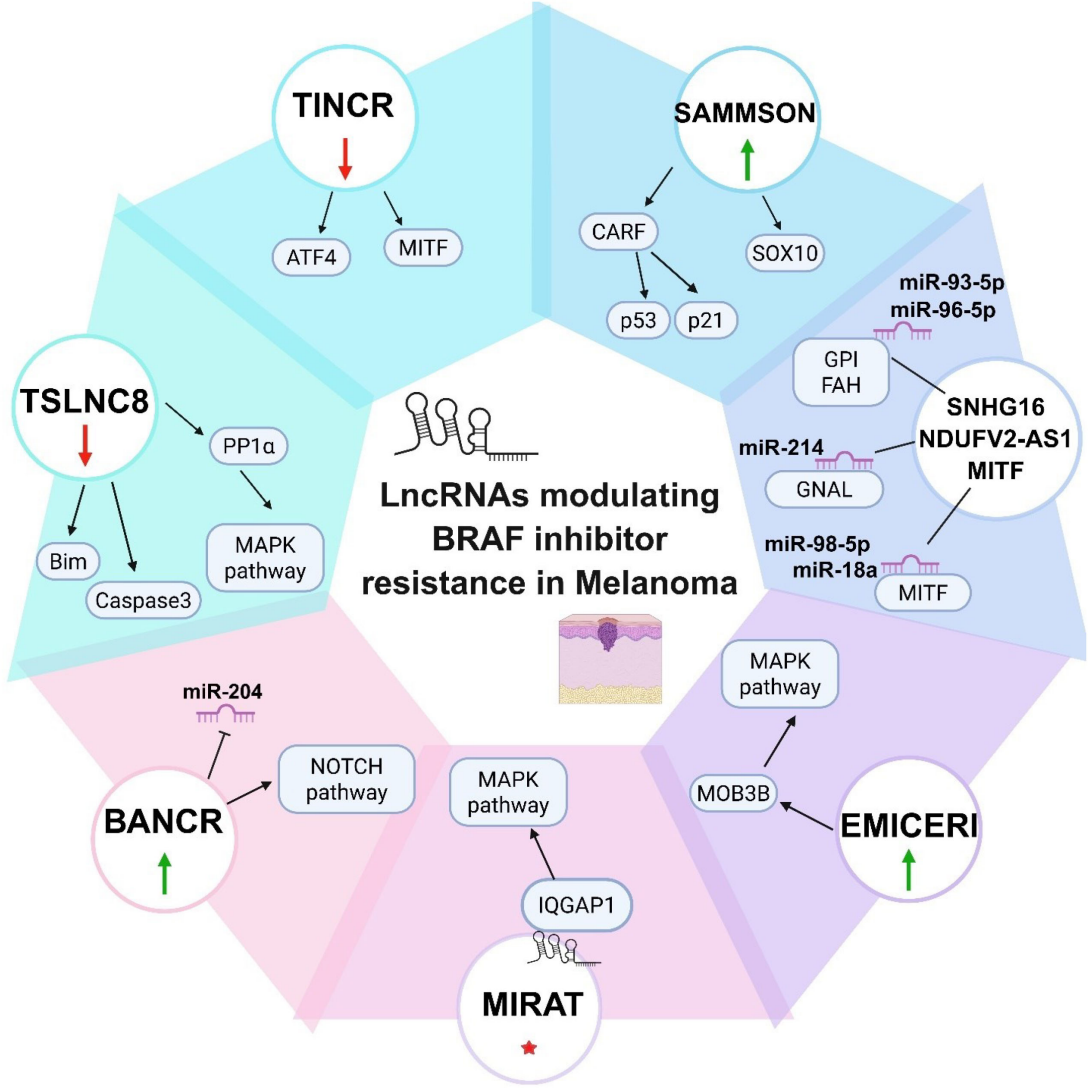
List of lncRNAs implicated in BRAFi-resistance in melanoma. Illustrations showing distinct lncRNAs that are known to regulate BRAFi resistance in melanoma. The visuals include the names of the lncRNAs in circle. Red arrow in the upside down position indicates down regulation, green arrow in the up position indicates up-regulation, and red color star indicates increased cytoplasmic localization.

BRAF is one of the most influential oncogenes in melanoma. Its mutations are not isolated event; rather they cause the MAPK pathway to become persistently activated, which promotes unchecked cell proliferation and significantly contributing to melanoma progression (2). Reactivation of the ERK pathway and BRAFi inefficiency are caused by overexpression of mutant BRAF, which favors dimerization (66). Because BRAFi primarily inhibit monomeric BRAF, their effectiveness is diminished when splice variants of BRAF^V600E^, such as p61BRAF^V600E^, can generate dimers independently of RAS. Furthermore, activation of other RAF isoforms, including ARAF and CRAF, contributes to BRAFi resistance, as pan-RAF isoforms can compensate for BRAF inhibition (67). The PI3K/AKT pathway’s activation is another significant pathway activated in resistance to BRAFis (66). In addition to these conventional melanoma resistance mechanisms, new preclinical studies have demonstrated the critical role for lncRNAs play in driving treatment resistance, especially in response to BRAFis. LncRNAs contribute to BRAFi resistance by regulating multiple aspects of drug response, including drug metabolism, transport, and cell survival pathways. They also interact with the chromatin-modifying complexes to influence epigenetic regulation of gene expression and affect key molecular pathways, like MAPK/ERK pathway, which is directly targeted by BRAFis. As shown in **Figure 4** and in the following sections we have discussed them in detail.

### 5.1 Major lncRNAs involved in BRAF inhibitor resistance

Numerous genomic and transcriptomic investigations have demonstrated a strong correlation between melanoma and lncRNA dysregulation. One study, analyzing 7,256 RNA sequencing libraries across 27 different tissue types, identified 339 lncRNAs specifically linked to melanoma (68). To investigate whether lncRNAs are regulated by mutant BRAF in primary melanomas, Flockhart and colleagues conducted transcriptome analyses following ectopic expression of the BRAF^V600E^ mutation in normal melanocytes. This study identified 39 lncRNAs and 70 novel non-coding transcripts induced by mutant BRAF (69). Among them, a previously uncharacterized lncRNA, BANCR, was found to be differentially expressed in primary melanomas compared to normal melanocytes (69). Functionally, silencing BANCR led to elevated levels of CXCL11, a chemokine known to inhibit MAPK signaling and suppress tumor growth and migration (69). Moreover, miR-204 is competitively regulated by BANCR, which functions as a competing endogenous RNA (ceRNA) and has been associated with poor prognosis in melanoma (70). Similarly, Goedert et al. demonstrated a strong correlation between lncRNA RMEL3 and the BRAF^V600E^ mutation, suggesting that RMEL3 may contribute to melanoma cell survival and proliferation (71). Additionally, the authors showed that silencing RMEL3 led to increase levels of the tumor suppressor PTEN, which frequently prevents BRAF^V600E^ driven oncogenesis, while simultaneously decreasing BRAF levels. RMEL3 knockdown consistently produced a strong inhibition of BRAF^V600E^ melanoma cell proliferation and survival (71). Together these studies raise an important question as to what extent do lncRNAs contribute to BRAFi resistance in melanoma. In the following subsections, we provide a detailed description of lncRNAs that have directly implicated in BRAFi resistance in melanoma.

#### 5.1.1 EMICERI

To identify noncoding loci that influence BRAFi resistance phenotype, Joung et al. developed a genome-scale CRISPR-Cas9 activation screen targeting more than 10,000 lncRNA transcriptional start sites. They discovered 11 new lncRNA loci that each mediate BRAFi resistance in melanoma when an activator is recruited (72). Their investigation of both local and non-local mechanisms suggests that most of these putative loci regulate neighbouring genes. A comprehensive assessment of one candidate, known as EMICERI, showed that its transcriptional activation induces the expression of four nearby protein-coding genes (EQTN, MOB3B, IFNK, and C9orf72) in a dosage-dependent manner, one of which is directly implicated in the resistance phenotype (72). By demonstrating that activation of EMICERI/MOB3B axis conferred vemurafenib resistance in two additional BRAFi-sensitive melanoma cell lines and by showing its association with a vemurafenib-resistant gene-expression signature in melanoma patients from TCGA, the authors concluded that the EMICERI–MOB3B regulatory axis plays a decisive role in mediating vemurafenib resistance (72).

#### 5.1.2 SNHG16/NDUFV2-AS1/LINC01502

To identify novel annotated lncRNAs potentially involved in resistance to BRAFis, Shamloo et al. examined lncRNA expression in two BRAFi-R clones derived from SKMEL-239 melanoma cell lines. They performed RNA-Seq and ChIP-Seq analyses in both BRAFi-sensitive and BRAFi-R SKMEL-239 cells and integrated these datasets using an automated *in-house* pipeline, termed lncRNA-Screen (73). This method made it possible to identify the 162 lncRNAs that were significantly overexpressed in resistant cells compared to their sensitive counterparts. Notably, their focused CRISPRa screen validated 15 of the 162 lncRNAs which are similar in number to the study by Joung et al, functionally linking the role of lncRNAs to the BRAFi resistance phenotype (73). More recently, employing a comparable strategy, Wen et al. identified three BRAFi resistance–associated lncRNA genes, SNHG16, NDUFV2-AS1, and LINC01502, using a genome-scale transcriptional activation assay combined with a CRISPR-Cas9 synergistic activation mediator (SAM) system (74). The expression levels of these three lncRNs exhibit a strong positive correlation with the IC_50_ value for dabrafenib across melanoma cell lines, suggesting their involvement in BRAFi resistance. To further explore their functional significance, the researchers constructed a lncRNA–miRNA–mRNA network consisting of 13 nodes: three lncRNAs, six miRNAs, and four mRNAs. Network analysis showed a tightly connected regulatory axis, with strong positive associations among its components. These lncRNAs also exhibited differential expression in melanoma patient samples and participate in regulatory networks involving metabolism- and signaling-related mRNAs. In particular, SNHG16 positively correlates with GPI and FAH, which are functionally linked to mTOR activation, ERK/MAPK signaling, and metabolic reprogramming—pathways known to contribute to BRAFi resistance. Notably, three up-regulated mRNAs (MITF, FAH, GPI) within this network were found to be downstream targets of the candidate lncRNAs and were linked to BRAFi resistance based on Kaplan-Meier survival analysis (74). Furthermore, elevated expression of MITF and FAH was strongly associated with poorer prognosis in patients harboring BRAF mutations.

#### 5.1.3 SAMMSON

Ghasemian et al. have extensively reviewed the carcinogenic role of SAMMSON in the progression of melanoma (75). Focal amplifications of chromosomes 3p13–3p14, a particular site of SAMMSON, are present in about 10% of melanomas and are linked to a poor prognosis. The melanoma-specific oncogene MITF is located in the centre of this amplicon. The epicentre of this amplicon1 is home to the melanoma-specific oncogene MITF. Studies in the past have shown that the SAMMSON lncRNA is consistently co-expressed with MITF (40, 76). According to every study conducted to date, SAMMSON is a target of the lineage-specific transcription factor SOX10 and is expressed in more than 90% of human melanomas. Whether the melanoma cells have a BRAF, NRAS, or TP53 mutation, or whatever transcriptional cell state they are in, SAMMSON knockdown significantly reduces their survivability (40, 76). Using *in vitro* and patient-derived xenograft models, Leucci et al. demonstrated that SAMMSON targeting makes melanoma more sensitive to MAPK-targeted treatments. The mechanism by which SAMMSON increases its pro-oncogenic activity and mitochondrial targeting is through its interaction with p32, a master regulator of mitochondrial metabolism and homeostasis (40). Regarding the concern of BRAFi, Han et al. reported that SAMMSON was upregulated as early as 6 hours following the exposure of mutant BRAF melanoma cells to ERK signaling inhibitors. Their study demonstrated that exogenous expression of SAMMSON protected melanoma cells from vemurafenib-induced apoptosis, whereas SAMMSON depletion enhanced cell death in response to the drug (77). Mechanistically, they identified SOX10 as a critical modulator of vemurafenib-induced SAMMSON expression, with sumoylation at lysine 55 (K55) playing a critical role in this regulatory process. Furthermore, SAMMSON was shown to control the nuclear-cytoplasmic translocation of CARF, a function believed to influence p53 stability. Notably, knockdown of either CARF or p53 reversed the enhanced anti-tumor effects of vemurafenib observed upon SAMMSON depletion—both *in vitro* and in a mouse xenograft model, highlighting the functional significance of this regulatory axis in BRAFi resistance (77).

#### 5.1.4 MIRAT

A long intergenic non-coding RNA called MIRAT has been linked to the development of cancer, specifically in melanoma that has NRAS and BRAF mutations. In contrast to any other lncRNAs, MIRAT was detected in the cytoplasm of melanoma cells, particularly those that are resistant to MEK inhibitors, and is situated on chromosome 8q24.12 (18, 78). According to the study by Sanlorenzo et al, MIRAT promotes MAPK pathway signaling by attaching itself to the scaffold protein IQGAP1, stabilizing it, and preventing its degradation. IQGAP1 plays a crucial role in facilitating signal transduction from MEK to ERK. Prior research has demonstrated that IQGAP1 is increased in several tumors and could be a promising therapeutic target for the treatment of melanoma and other cancers with hyperactivated MAPK signaling (78). Through microarray profiling, the authors further found that after MIRAT knockdown, there was a predominance of upregulated transcripts in both the tested cell lines. Importantly, pathway enrichment analysis of these transcripts indicated an overrepresentation of genes associated with cell surface receptor–linked signal transduction, consistent with a role for MIRAT in modulating signaling networks rather than directly driving cell viability changes (78). This pattern of gene expression changes may reflect the engagement of signaling pathways that compensate for reduced MAPK signaling output upon MIRAT depletion, though the original study did not provide a comprehensive list of individual upregulated genes. Although the authors did not directly investigate the physiological role of MIRAT in BRAFi-resistant cells, one might argue that MIRAT is not a direct mediator of BRAFi resistance but rather a contextual modulator of MAPK signaling.

#### 5.1.5 TINCR

Human chromosome 19p13.3 contains the lncRNA TINCR, sometimes referred to as placenta-specific protein 2 (PLAC2), which is aberrantly expressed in a variety of human malignancies. Sharma et al. have extensively reviewed the pathogenic involvement of TINCR in various cancer types (79). For instance, in gastric cancer, TINCR prevented apoptosis by targeting the KLF2 gene (80). Similar conclusions were also clarified, indicating a strong correlation between TINCR levels and the regulation of the PI3K/AKT/mTOR signaling pathways that contribute to colorectal carcinogenesis through the sponging of miR-7-5p (81). According to the study by Melixetian et al. the lncRNA TINCR is downregulated in metastatic melanoma. In contrast, nevi-like initial melanomas exhibit noticeably enhanced levels of TINCR expression, which may contribute to the maintenance of a non-invasive characteristic. Functional studies demonstrated that TINCR silencing in proliferative phenotype melanoma cell lines induced a phenotypic switch towards invasiveness, as indicated by the increased expression of conventional invasion markers, enhanced *in vitro* migration, and resistance to both BRAFi and MEKis (18). Conversely, TINCR overexpression resulted in decreased *in vitro* cell migration and *in vivo* metastatic potential. Mechanistically, the authors found phosphorylated EEF2 levels were reduced by TINCR silencing, but the ATF4 protein and its transcriptional program were significantly up-regulated. Overall, the findings from the study suggested that TINCR is involved in regulating the invasive and drug-resistant phenotype of melanoma cells (18).

#### 5.1.6 TSLNC8

The carcinogenic significance of SAMMSON in the development of cancer, particularly melanoma, has been thoroughly studied by Li et al. (82). Long Intergenic Non-Protein Coding RNA 589 (LINC00589) or Chromosome 8 Open Reading Frame 75 (C8orf75) are other names for Tumor Suppressor LncRNA on Chromosome 8p12 (TSLNC8), which is found on Chromosome 8p12 and consists of four exons that do not overlap with designated coding genes. Altered expression of the lncRNA TSLNC8 has been reported across multiple cancer cell lines and tumor tissues (83, 84). By taking part in various tumor-related processes, TSLNC8 regulates drug resistance and aids in the initiation and spread of several human malignancies (82, 85). Given its dysregulation across a range of malignancies, and its significant associations with patient survival and clinicopathological features, TSLNC8 appears to be a functionally relevant and clinically significant lncRNA (82, 85, 86). Among the various studies, Han et al. provided direct evidence that BRAFi-resistant tissues and cell lines exhibit a marked downregulation of the lncRNA TSLNC8. Additional assay results showed that TSLNC8 downregulation prevents BRAFi-sensitive cells treated with BRAFi (PLX4720) from undergoing apoptosis. TSLNC8 can mechanistically bind to the protein PP1α, and when TSLNC8 is downregulated, PP1α accumulates in the cytoplasm and MPK signaling is further activated. Most importantly, TSLNC8 upregulation in BRAFi-resistant cells restores the inhibitor’s effect (85). Furthermore, in an *in vivo* experimentation, the authors found that overexpression of TSLNC8 enhances the cytotoxic effect of BRAFi as assessed by the reduction in tumor growth rate.

## 6 Conclusion and future directions

**Figure 5:**
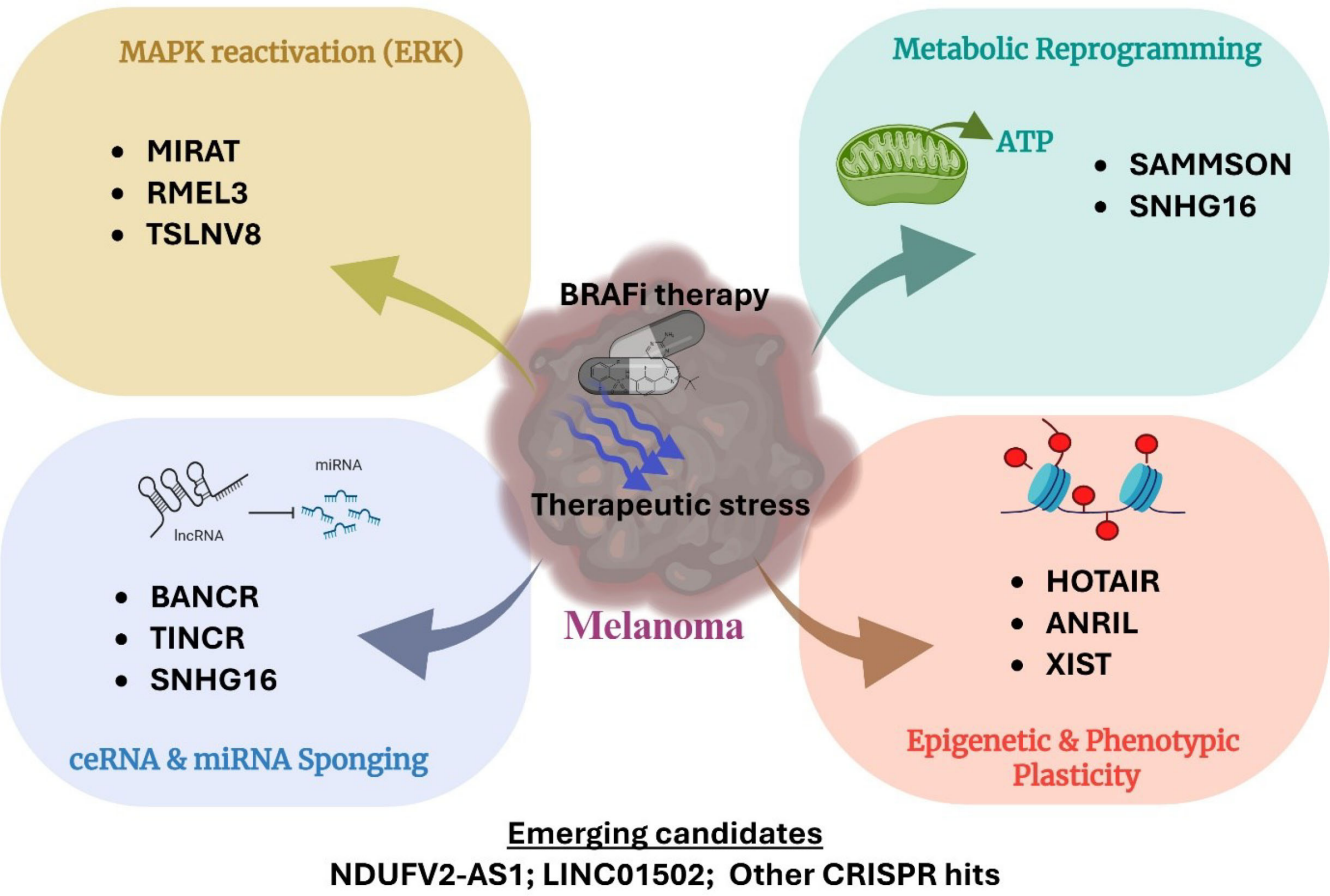
Functional convergence of lncRNAs driving BRAF inhibitor resistance in melanoma. Illustration showing how mechanistically diverse lncRNAs converge on shared biological processes that promote resistance to BRAFi. LncRNAs are categorized based on their roles in MAPK pathway reactivation, metabolic reprogramming, post-transcriptional regulation via ceRNA networks, and transcriptional plasticity. Collectively, these convergent mechanisms enable melanoma cells to adapt and survive under BRAFi–induced stress.

Despite significant developments in therapies targeting mutated BRAF, the MAPK pathway, and immune checkpoints in melanoma, low response rates and acquired resistance to these options remain the leading causes of recurrence and patient mortality. Although research on the role of lncRNAs in chemoresistance is still in its early stages, growing evidence suggests that they may serve as molecular targets for future therapies. The present review discusses the mechanism of action behind the control of essential signaling pathways, as well as the significant biological roles of various lncRNAs in BRAFi-resistance in melanoma (**Figure 4**). The conceptual framework presented in **Figure 5** highlights how mechanistically distinct lncRNAs converge on a limited number of core resistance pathways, underscoring their potential as integrative therapeutic targets. Before lncRNA-based cancer treatments or biomarkers can be used in clinical settings for BRAFi-resistant melanoma, we believe that much more study is required. Currently, BRAFi-resistance in melanoma is a complex biological process where little is known about the functions and mechanisms of lncRNAs. Most research is conducted in *in vitro* systems, rendering *in vivo* studies essential for gaining meaningful functional insights. Nevertheless, creating a model of lncRNA function in mice remains challenging. An important point to consider is that, compared to protein-coding genes, lncRNAs exhibit significantly lower sequence conservation. As a result, lncRNAs that have been effectively validated in animal models might not be suitable for use in clinical settings. As discussed in the present review, state-of-the-art tools like CRISPR-Cas9 and single-cell sequencing have been instrumental in uncovering the role of lncRNA in BRAFi-resistance. CRISPR-based methods, such as CRISPR activation (CRISPRa) and CRISPR interference (CRISPRi), provide effective means of selectively silencing or activating lncRNA loci, allowing for the causal evaluation of their roles in drug resistance while maintaining endogenous genomic context. Tumor heterogeneity and cellular plasticity are two more significant limitations that bulk transcriptomic analyses are unable to fully capture. Rare drug-tolerant subpopulations and adaptive transcriptional states linked to BRAFi resistance, such as lncRNAs associated with invasive and MITF-low phenotypes, have started to be discovered by single-cell RNA sequencing. It may be possible to directly connect lncRNA activity to cellular states linked to resistance by integrating single-cell data with functional perturbation platforms like Perturb-seq. When combined, these strategies offer a path forward for overcoming present constraints and moving lncRNA research in the direction of mechanistic understanding and translational significance in BRAFi-R melanoma. We believe that greater priority should be given to identifying lncRNAs involved in resistance to recently approved BRAFi, such as Encorafenib, or combination regimens involving MAPK inhibitors and Encorafenib, before resistance to these therapies also emerges.

However, currently several challenges exist that limit their clinical translation as they often exhibit strong tissue- and context-specific expression, and many are expressed at low abundance, complicating their reliable detection in circulation. Furthermore, it remains difficult to distinguish tumor-derived lncRNAs from background transcripts released by normal tissues. Circulating lncRNAs, which can be detected through non-invasive liquid biopsies, seems to be useful biomarkers for monitoring the acquisition of resistance. But the stability of circulating lncRNAs, variability in pre-analytical sample processing, and lack of standardized RNA isolation and quantification protocols represent major obstacles. While exosome-associated lncRNAs may offer improved stability, methodologies for their isolation and analysis remain heterogeneous. In the age of precision oncology, the clinical development of lncRNA-based diagnostics and therapeutics requires rigorous functional validation, large-scale longitudinal studies, and regulatory standardization. Addressing these challenges will be essential for translating lncRNA discoveries from bench to bedside and realizing their potential in precision melanoma therapy.
